# A comparative cohort study for detecting the incidence of trisomy 21 in ART and non-ART neonates 

**Published:** 2014-06

**Authors:** Ataollah Ghahiri, Amin Firozmand, Mojdeh Ghasemi, Fahime Nasiri, Maryam Sharifi, Mehry Abdollahi

**Affiliations:** 1 *Department of Obstetrics and Gynecology, Isfahan University of Medical Sciences, Isfahan Iran.*; 2 *Isfahan University of Medical Sciences, Isfahan, Iran. *; 3 *Shahid Beheshti Hospital. Isfahan University of Medical Sciences, Isfahan, Iran.*; 4 *Azahra Hospital, Isfahan University of Medical Science, Isfahan, Iran.*

**Keywords:** *Infertility*, *trisomy 21*, *ART*, *neonate anomaly*

## Abstract

**Background:** One of the most important points concerning the patients who undergo assisted reproductive techniques (ART) for getting pregnant can be the possible neonatal chromosomal abnormalities as a result of these methods.

**Objective:** This study was conducted to help answering a part of this question.

**Materials and Methods: **This is a historical cohort study from April 2006 to April 2007. Data were collected from women admitted in Mehregan Hospital and Esfahan Infertility Center. 225 of 2000 infertile women who had taken ART methods and 225 of 1800 women undergoing no ART treatment were included in our study. All of the cases were aged 35 or more. Data were obtained from patient files from 2 infertility centers of Isfahan, Iran.

**Results: **Chromosomal analysis was successfully performed for all clinically suspicious infants for trisobmy 21. As a result, 4 cases of trisomy 21 in ART group and 7 in non-ART group were found. Two cases from IUI, 1 case of IVF and 1 of ICSI were found to have trisomy 21 in infants. There was no statistically difference in occurring trisomy 21 in our two groups of study and this was also the same for women undergoing IVF and ICSI.

**Conclusion:** ART methods did not increase the rate of Trisomy 21 according to our study although we found less in ART group, it was not statistically significant.

## Introduction

Chromosomal abnormalities such as trisomy 21 is one of the most important problems concerning the mothers who seek pregnancy. Is it more common in neonates coming from assisted reproductive techniques (ART) in the patients with infertility? The study was conducted to answer this question. Peschka and colleagues showed that "a high number of infertile couples in an Intra cytoplasmic insemination ICSI program are affected by chromosomal aberrations which occur in both sexes" (1). In one study a decrease in pregnancy associated plasma protein A and an increase in HCG levels indicated more risk for occurring trisomy 21 after using ART methods (2). Feng *et al* for the first time, showed that risks of gene mutation may increase in the ART offspring, even though their fathers have normal spermatogenesis and genetic background (3). 

Screening for genetic factors and chromosomal abnormalities is indicated for couples undergoing ART due to the higher prevalence of these factors in infertile couples compared to the population as a whole although different chromosome aberrations have been reported elsewhere (4). Occurring chromosomal abnormalities in infants proportionally pertains to maternal age and highly influenced by the age of 35 or more. Some cases of trisomy 21 were reported in Isfahan infertility centers as a result of ART treatments especially in women by the age of 35 or more. This made a serious question in their mind to know if these methods really increase the incidence of trisomy 21 in their infants. Very few investigations had been made in Iran in order to offer a proper answer to this critical question. Different results reported by many studies motivated us to conduct a study to determine the prevalence of trisomy 21 at infants of women undergoing ART and compare it with who become pregnant by traditional methods.

## Materials and methods

In order to investigate the effect of ART on occurring trisomy 21 in infants, we run a historical cohort study from April 2006 to April 2007. The place for our study was Isfahan Infertility Center and Mehregan Hospital that both were assumed as important centers in this field. Information was taken from patients’ files that were existed in our centers of reference. Oral consent was obtained from participants by phone. This article has been accepted as a student thesis in Isfahan University of Medical Sciences and ethical committee by this code: 387451.

The including criteria was women 35 years old or more who underwent ART treatments because of infertility and made delivery in our centers of reference and also women that were not under the mentioned treatments by the same range of age that came to the centers for the same reason as the control group. Tests were done for all clinically suspicious infants for diagnosing trisomy 21. The exclusion criteria were those infertile women who delivered neonates with other anomalies and those patients aged under 35. Genetic tests for trisomy 21 were just obtained for clinically suspicious infants that included dysmorphic features, congenital malformation, and other health problems and medical condition. 225/2000 women by the age of 35 and beyond had ART from April 2006 to April 2007 were collected by the mentioned methods.

225 of 1800 women by the age of 35 and beyond without taking ART who came to the same centers for delivery were selected by the same method during the same time. All of the cases and also the control group were examined during one complete year. Data were collected from structural check lists that were existed for all women in our study. Cases of trisomy 21 in infants were collected by the files that were in the same centers. The rates of trisomy 21 in infants as a result of In Vitro Fertilization (IVF) and ICSI were also compared. Chromosomal studies to detect trisomy 21 were also available at the same centers by genetic specialists. Tests were performed for all clinically suspicious neonates who were reported by neonatologists.

**Statistical analysis**

X^2^ Test and Fisher, S exact test were applied for statistical evaluation in order to compare the prevalence of trisomy 21 between ART and non-ART group. Data were analyzed by SPSS software (version 15).

## Results

As a result 225 women in ART group by the mean age of 37.04±2.43 years were compared with 225 women in the normal group by the mean age of 37.13±2.44 years. Leven’s test for equality of variances and t-test for equality of means were performed. The ART group consisted of IUI (intra uterine insemination), IVF (invitro fertilization) and ICSI (intracytoplasmic injection). According to age description 110 cases of IUI in our study had a mean age of 37.22 years and 57 cases of IVF had 36.75 years and 58 cases of ICSI had 36 years respectively. In non-ART (normal) group a mean age of 37.13 years was observed. The mean age of the total group that consisted of all 450 women was 37.08 years which is shown in Descriptive table. The two groups were demographically matched. The normal group consisted of 7 cases of trisomy 21 (3.1%) and 218 infants remaining without diagnosing of trisomy 21 (96.9%). 

In ART group 4 cases of trisomy 21 (1.8%) were existed and the remainder accounted for 221 infants (98.25%). The total number of trisomy 21 was 11 cases (2.4%). Trisomy 21 percentile in both groups is shown in table I. The rate of trisomy 21 in ART group accounted for 2 cases (1.8%) in IUI and 1 case (1.8%) in IVF and 1 case (1.7%) in ICSI (Table II). There was no statistically difference between the two above mentioned groups. There was no significant difference in occurring trisomy 21 between IVF, IUI and ICSI. 

**Table I:** Comparison of the rate of trisomy 21 in ART and non-ART groups

	**Trisomy 21**	**Normal neonates**
ART group^*^	4 (1.8%)	221 (98.2%)
Non ART group^*^	7 (3.1%)	218 (96.15)
Total	11	439

Data are presented as n (%).

**Table II:** Rate of trisomy 21 in different type of ART

	**IUI**	**IVF**	**ICSI**	**Total**
ART group	2 (1.8%)	1 (1.8%)	1 (1.7%)	4 (1.85)

Data are presented as n (%).

## Discussion

The infertile people who are candidate for ART management always are worry about the probability of developing congenital anomalies in their infants if they become pregnant. Trisomy 21 is the most important anomaly between the live neonates (5). Some studies have shown different results about this problem. As a beginning we conducted this comparison study just for investigating the prevalence of trisomy 21 in infants of ART and non-ART groups. It is also difficult to find out and compare the rate of trisomy 21 in ICSI, IVF and IUI in literature (6). 

As mentioned above, there was no significant difference of trisomy 21 in infants between these two mentioned groups (ART and non-ART group). There was no significant difference in occurring trisomy 21 between IVF and IUI and ICSI respectively. Many studies in this field confirm our findings to some extents. In the study of Simpson and lamb malformations in live born infants were not increased as a result of ICSI (7). Basaran *et al* indicate that cytogenetic investigations of the ICSI parents and fetuses are essential for the families, genetic counselors and also reproductive centers. In fetal karyotyping, de novo structural chromosome abnormalities and mosaicism should be taken into consideration (8).

Neri *et al* showed there was no indication that ICSI children present more congenital defects or express a lower psychomotor development (9). In another study higher prevalence of trisomy 21 was not detected as a result of ICSI (7). Macas *et al *showed that the incidence of chromosomal anomalies in fetuses resulted from ICSI was more than in IVF (10). In a study in Lebanon, results show significantly higher frequencies of sex chromosome disomies in the group of patients with oligozoospermia compared with a control group of fertile males. Altogether, the results suggest that Lebanese oligozoospermic men undergoing ART may have an increased risk of transmitting sex chromosome anomalies to their offspring, as well as, in some cases, trisomy 21 (11). 

Morel’s *et al* stressed the importance of karyotyping both male and female partners before ICSI. Adequate genetic counselling, possibly followed by prenatal diagnosis, should be offered if a chromosomal anomaly is detected (12). Couples should be informed of the risks of an abnormal result related to sperm quality, and of the risk linked to a prenatal procedure as well as about the relatively benign character of some chromosomal anomalies such as de-novo structural anomalies or sex chromosomal anomalies in order to be able to make a choice for prenatal testing, or not (13). We cannot propose a precise and complete result because of the limitations of our study. For instance trisomy tests were just made for the clinically suspicious infants who may affect our results.

## Footenote

***This article extracted from M.D. thesis. (Amin Firouzmand)***
